# Fatigue Test of 6082 Aluminum Alloy under Random Load with Controlled Kurtosis

**DOI:** 10.3390/ma14040856

**Published:** 2021-02-10

**Authors:** Robert Owsiński, Adam Niesłony

**Affiliations:** Faculty of Mechanical Engineering, Opole University of Technology, 45-758 Opole, Poland; a.nieslony@po.edu.pl

**Keywords:** fatigue tests, electromagnetic shaker, kurtosis

## Abstract

This paper presents the results of experimental tests carried out on an electromagnetic shaker where the excited element was a specimen with additional weight attached to the slip table. The load was random with a different kurtosis parameter value, i.e., it was performed for non-Gaussian loads. The experiment was accompanied by basic fatigue calculations in the frequency domain and their verification with experimental results. A significant decrease in fatigue life was found to take place with an increase in kurtosis and the maintenance of the same standard deviation of the specimen load. The fatigue effect, caused by the deviation from the normal distribution that was described by the kurtosis parameter, on the fatigue life of aluminum alloy 6082 was presented. An analysis revealed the different amplitude probability distributions for the loading signal and the recorded deformation signal. It was concluded that there was a lack of sensitivity of the numerical model to the change in the kurtosis parameter of the distribution of random loads.

## 1. Introduction

The loads of machine and device elements, which occur during typical work or operation and change over time, are usually not deterministic and can be treated as a random variable [1,2,3,4]. In such cases, it is reasonable to use tools from mathematical statistics and stochastics to estimate the parameters used in fatigue calculations [5,6]. In these calculations, parameters such as the expected number of passes through the zero level, the maximum signal value, the crest factor, the expected number of cycles of a load, etc., are helpful [7,8,9]. However, it should be remembered that these methods and parameters have certain limitations that narrow the scope of application of fatigue analysis. One of the significant limitations is the assumption that the distribution of instantaneous values of the load signal can be described by a Gaussian distribution [10]. However, mapping real loads is difficult due to the frequently occurring non-Gaussian signals. Therefore, in fatigue calculations, additional statistical parameters, such as kurtosis and skewness, should be used to account for the deviation in the load course from the Gaussian waveform [3,11,12,13].

Because the change in and subsequent control of the excitation signal kurtosis parameter is an effective way to shorten the testing time of machine and device elements, it is of interest to analyze how the fatigue damage experienced by the tested element changes for different values of this parameter [14].

The use of the kurtosis parameter when generating the signal’s time history based on the given random load spectrum influences the amplitudes’ value of the acceleration signal occurring during fatigue tests with random loads while maintaining the same Root Mean Square (RMS) values and the same Power Spectral Density (PSD) shape of the forcing signal. Moreover, the energy expenditure in such a test for different kurtosis values is near constant because the RMS is preserved [15,16]. The standard time history of a random load with a Gaussian distribution is changed by replacing the corresponding amplitudes in the middle range with large amplitudes (peaks) and amplitudes in the lower range with minimal accelerations (close to zero). Therefore, the total energy of the test performed remains the same, with a simultaneous increase in the number of peak accelerations, which affects the discussed shape of the acceleration amplitude distribution. Obviously, a change in the acceleration of higher amplitudes leads to higher local stresses in the tested elements, which accelerates the accumulation of fatigue damage and the appearance of cracks [17].

In order to determine the differences in the obtained fatigue life of the 6082 aluminum alloy at different kurtosis values of the loading signal and to understand the degradation phenomena, a number of experimental tests were carried out, supplemented with numerical analyses using the Finite-Element Method (FEM) and the analysis of the fatigue cracks obtained.

The aim of the article is to present the fatigue effects caused by deviation from the normal distribution described by the kurtosis parameter on the fatigue life. The authors of the article present the results of experimental tests performed on 6082 aluminum alloy along with the results of the accompanying analyses of surface quality measurements, fatigue damage, and the results of numerical tests. The conclusions are included in detail at the end of the article and indicate the need for continued research in this area.

## 2. Theoretical Basis and Equipment

The time history probability distribution of a Gaussian load can be described by a Gaussian distribution, also known as the normal probability distribution:

(1) $$f\left(x\right)=\frac{1}{\sigma \sqrt{2\pi }}\exp\left(\frac{-{\left(x-\mu \right)}^{2}}{2\sigma^{2}}\right),$$

which contains two parameters: mean value μ:

(2) $$\mu =E\left[X\right]=\underset{T\to \infty }{\lim}\frac{1}{T}\overset{T}{\underset{0}{\int }}x\left(t\right)dt$$

and variance $\sigma^{2}$:

(3) $$\sigma^{2}=E\left[{\left(X-\mu \right)}^{2}\right]=\underset{T\to \infty }{\lim}\frac{1}{T}\overset{T}{\underset{0}{\int }}{\left[x\left(t\right)-\mu \right]}^{2}dt.$$

For non-Gaussian loads, other distributions should be used to describe the probability density $f\left(x\right)$. These usually contain more than two parameters, and the mathematical forms for the distribution are far from normal. However, in the science of fatigue, the load signal is usually assumed to be Gaussian because it allows for the use of many formulas of great practical importance. Therefore, only the normal distribution is used, and deviation from this distribution is presented as additional statistical parameters used for various correction coefficients [10]. These parameters include skewness Sk

(4) $$Sk=\frac{\mu_{3}}{\sigma^{3}}$$

where $\mu_{3}$ is the third central moment determined according to the general formula for central moments of the order *k* of a discrete random variable

(5) $$\mu_{k}=E\left[{\left(X-\mu \right)}^{k}\right]=\overset{+\infty }{\underset{-\infty }{\int }}{\left[x-\mu \right]}^{k}f\left(x\right)dx$$

and $\sigma^{3}$ is the value of the standard deviation raised to the third power, where the standard deviation is defined as the square root of the variance

(6) $$\sigma =\sqrt{\sigma^{2}}$$

**Figure 1:**
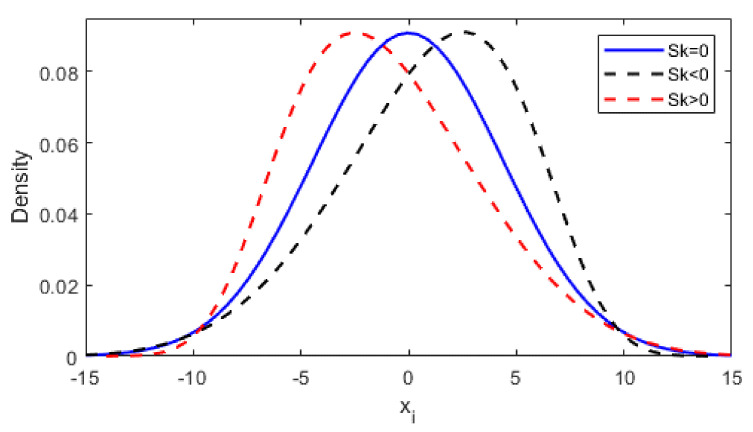
Distortion of the probability distribution for different values of the skewness parameter.

The skewness factor, Equation (4), describes the degree of asymmetry of the distribution. For Sk<0, we have a left-hand distribution; for Sk>0, the distribution is right skew, and for Sk=0, the distribution is symmetrical, as shown in Figure 1.

Another statistical parameter used to describe the shape of the distribution of a random variable is the concentration coefficient known as kurtosis, which is defined as the ratio of the fourth central moment (5) to the standard deviation (6) raised to the fourth power:

(7) $$Ku=\frac{\mu_{4}}{\sigma^{4}}$$

**Figure 2:**
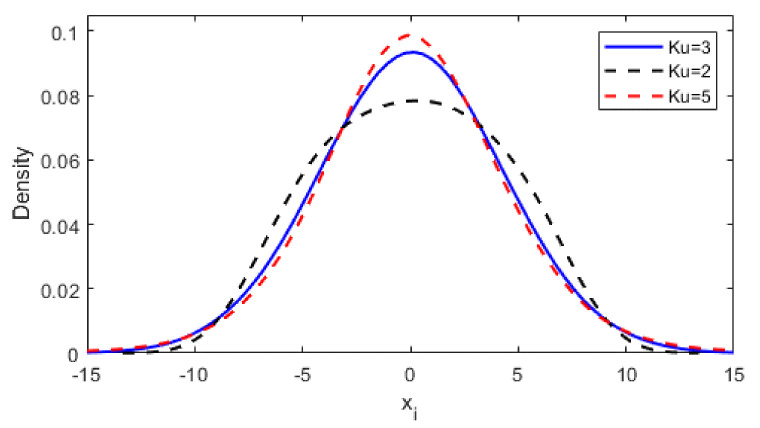
Change in the distribution of a random variable for different values of the kurtosis parameter.

Kurtosis is a measure of the so-called flattening of a distribution. The normal distribution (1) is characterized by a kurtosis value equal to Ku=3: this is a mesokurtic distribution. When Ku > 3, we say that the distribution is leptokurtic, and when Ku < 3, the distribution is defined as platykurtic. Figure 2 demonstrates the changes in the shape of the distribution described by kurtosis. With regard to fatigue analysis, it should be noted that it is possible to generate load curves for one PSD function profile with different values of the parameters Sk and Ku using the nonlinear transformation of the time history [2].

In laboratory practice, fatigue or durability tests are carried out on electromagnetic shakers while maintaining a given PSD load profile [18,19]. This is usually done by generating Gaussian load waveforms. It is known, however, that similar to the synthetically generated load histories and the observed real service loads, they may be of a non-Gaussian nature. Moreover, non-Gaussian load waveforms with different kurtosis and skewness parameter values may have the same PSD profile. This has been used to carry out endurance tests to achieve the effect of material fatigue in a shorter period of time or to reproduce the actual operating conditions of the device preserving the non-Gaussianity of the load. Hence, there was a need to model these issues and create computational algorithms that take into account the deviation from the normal distribution when estimating fatigue life using the spectral method.

### Experimental Setup

**Figure 3:**
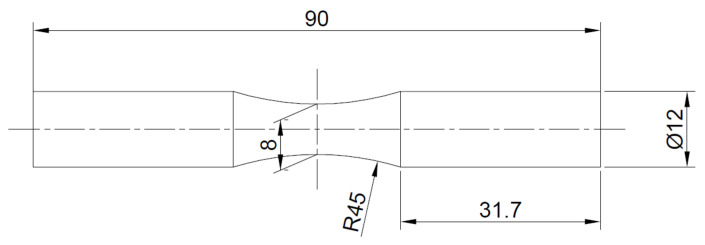
Specimen used in the experiments.

**Figure 4:**
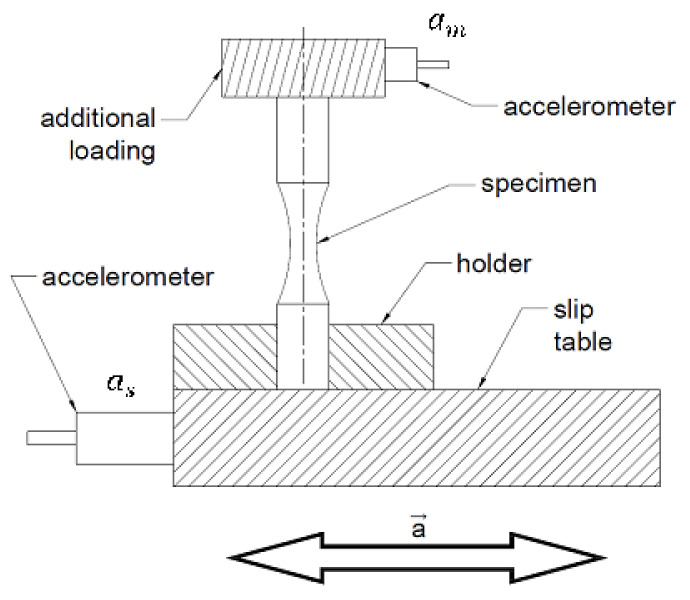
Fatigue test stand configuration with additional equipment.

The experimental tests were carried out on an electromagnetic shaker of the Dongling (Suzhou, Jiangsu Province, China) ET-3-150 type equipped with a GT300A slip table. Figure 3 shows a test specimen with its basic dimensions. The arrangement of the test system is shown in Figure 4.

The test program assumed that the fatigue tests were carried out under random load conditions. The random load was generated based on the PSD function in a rectangular shape with nonzero values in the range of 20–100 Hz. The load level was determined so that the specimen would break during testing in 1 to 8 h. Table 1, Table 2 and Table 3 show the load levels used during the tests. The tests were carried out until a full fatigue break was achieved or until the maximum assumed test time was reached: $t_{end}\;=\;8\;h$. The test was considered complete in the case of no fatigue break after 8 h of fatigue testing.

## 3. Fatigue Tests

A total of 31 specimens were subjected to fatigue tests. The identification of the dynamic response was carried out before the start of the test for each specimen with a mounted additional weight, as presented in Figure 4. On the basis of PSD shape and the measured test responses, the correct loading signal level was determined and controlled by the Siemens LMS TestLab 17 software. The handle of the sliding table was considered as being the point of load application and its course was measured there. The opposite end of the test specimen, at the point where the weight was attached, was where the test system response was measured. Before starting the tests, the surface quality of each specimen was checked with the MahrSurf PS 10 (Göttingen, Niedersachsen, Germany) contact profilometer.

**Figure 5:**
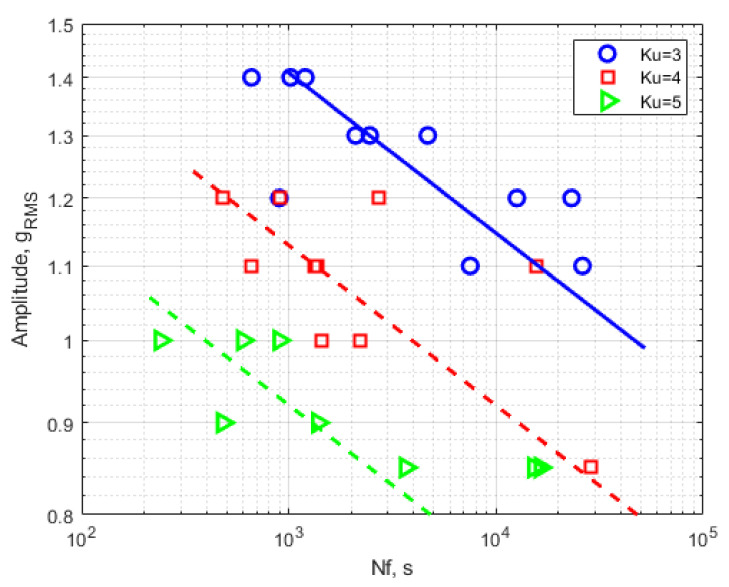
S–N curves for the three kurtosis values.

Table 4 summarizes the observed fatigue life of the experimental tests carried out for the assumed load levels listed in Table 1, Table 2 and Table 3. Figure 5 graphically presents the obtained results of the research in accordance with Table 4. The designations in the table regarding the type of single-sided and double-sided crack, the influence of the forcing signal parameters on the type of crack, and possible deviations from the fatigue life obtained are discussed in more detail in Section 3.3.

**Figure 6:**
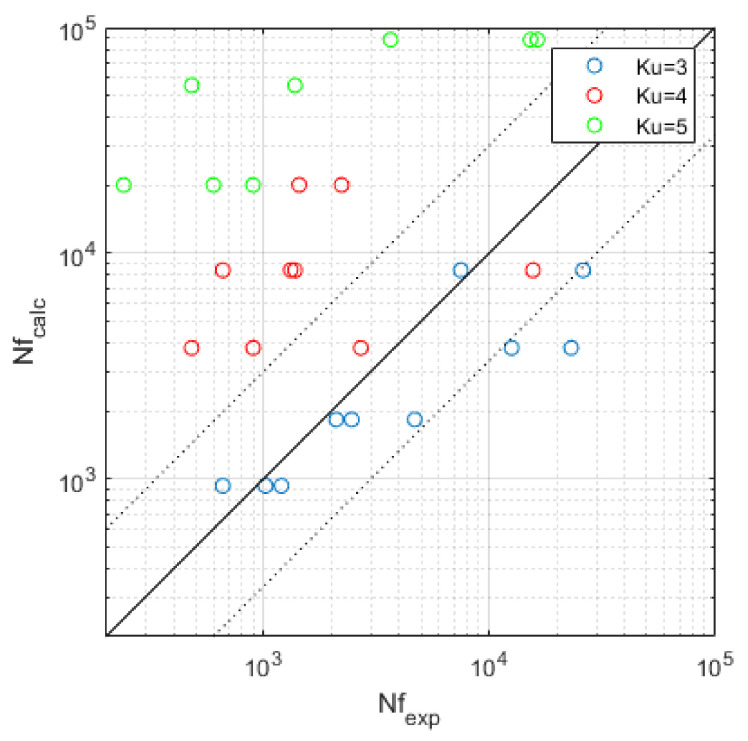
Comparison of experimental and predicted fatigue life.

**Figure 7:**
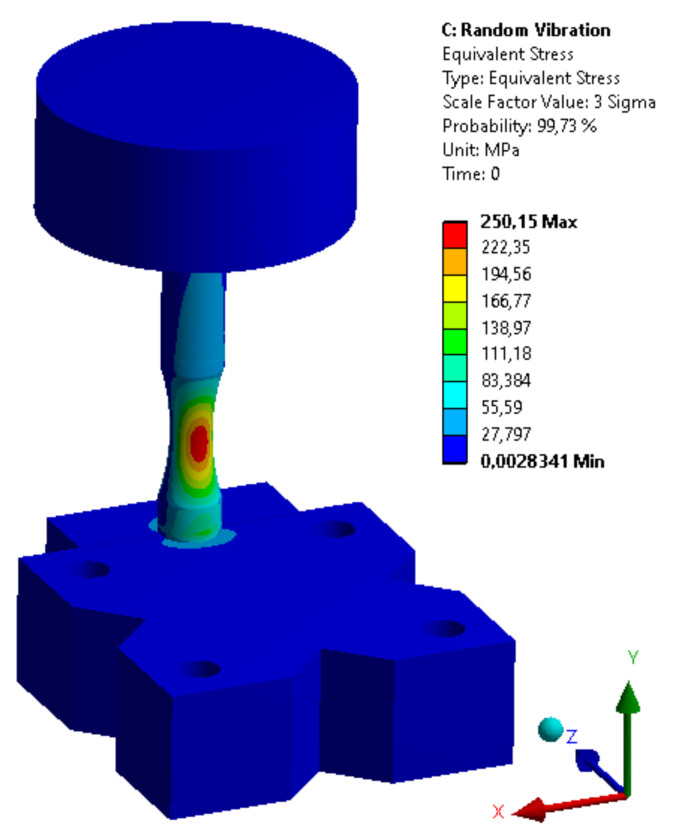
Numerical model used for fatigue analysis for the Ansys Workbench.

FEM calculations were performed with the Ansys Workbench 2020 R2 software. The regression coefficients for the material model adopted for numerical calculations of the following form of fatigue life:

(8) $$logNf=A+m\log\sigma_{a},$$

are A=20.7 and m=9.85, respectively [20,21]. The procedure for determining the predicted fatigue life of elements subjected to random loads determined by Acceleration PSD uses information based on the Steinberg model and the S–N characteristic of the material, determined in the conditions of cyclic loads based on parameters A and m (Equation (8)). As a result of the analyses carried out with the use of the Random Vibration module, forecasts concerning the expected fatigue life were obtained. The obtained results were compared with the experimental data in Table 5 and presented graphically in Figure 6. Figure 7 shows the numerical model used for the random vibration fatigue analysis.

The parameters of the calculation model in Ansys Workbench are summarized in Table 6 below.

### 3.1. Modal Analysis

The occurrence and form of natural vibrations were identified and confirmed numerically. A series of numerical analyses were carried out to identify the form and frequency of natural vibrations in the test system and to relate the obtained quantities to the data from the experiment. The results of these analyses are presented in Table 7.

**Figure 8:**
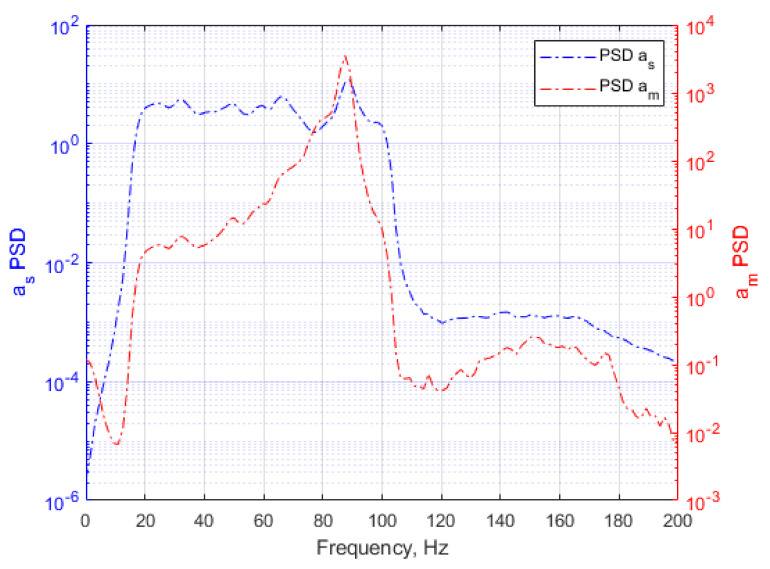
Power Spectral Density (PSD) for acceleration signals recorded during the test; kurtosis Ku = 3.

As a result of the symmetry of the specimen geometry and the symmetrical mounting of the additional mass, various vibration modes are similar, i.e., the first and second vibration modes and the fourth and fifth vibration modes. Similar natural vibration shapes differ in rotation by 90 degrees. The vibrational frequencies are similar and are about 90 Hz for Modes 1 and 2 and about 1015 Hz for Modes 4 and 5. The fatigue tests carried out on the test stand are characterized by the presence of one resonant frequency within the range of excitation frequencies from 20 to 100 Hz, which was the first mode of vibrations at 90 Hz. Figure 8 presents the acceleration PSDs of the input $a_{s}$, measured at the specimen $a_{m}$.

### 3.2. Strain Measurements on Specimens

**Figure 9:**
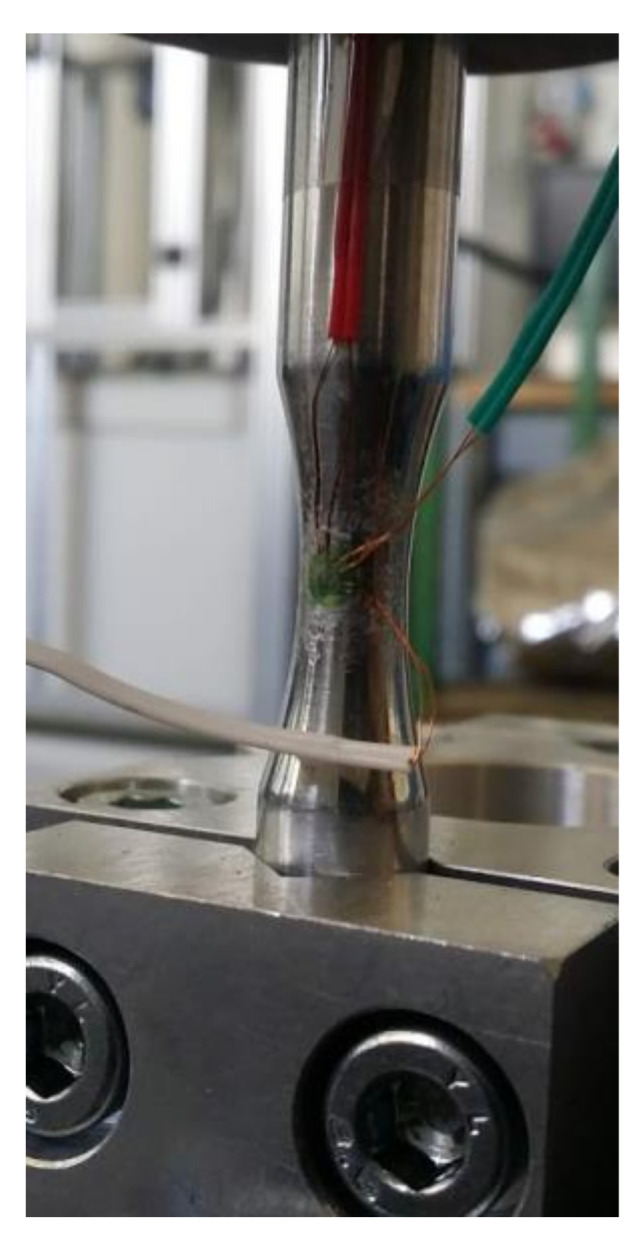
Specimen with a strain gauge for strain measurements during the experimental procedure.

**Figure 10:**
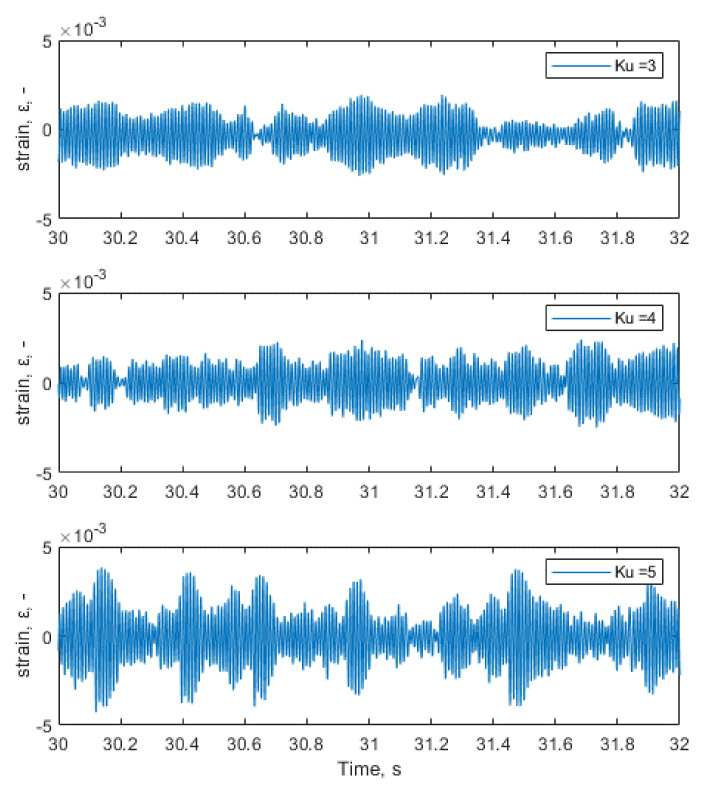
Strain time histories in specimens’ neck for different loading signal kurtosis values.

A procedure for identifying the experimental deformation value of the measuring part of the tested element was carried out on the presented test system (see Figure 9 for the location of the resistance strain gauge rosette). All measurements were performed on an appropriately prepared short test program lasting 60 s in order to obtain a representative data sample for further analysis, which would be repeatable for all realized loading conditions. Figure 10 shows a two-second portion from the recorded strain signal of the short test program for three different loading kurtosis values, in which it is easy to see that the amplitudes are distributed in different ways and in which greater amplitudes for higher kurtosis values are present. The deformations were recorded using a rectangular TML strain gauge rosette connected to a measuring system built using the National Instruments measuring device and a program prepared in the LabVIEW 2017 environment [22]. When analyzing the recorded deformation time signals, the kurtosis parameter value for such a recorded signal was examined. It was noted that for the recorded signal, the kurtosis differs from the kurtosis set on the excitation and was in the range of 2.5–3.2.

**Figure 11:**
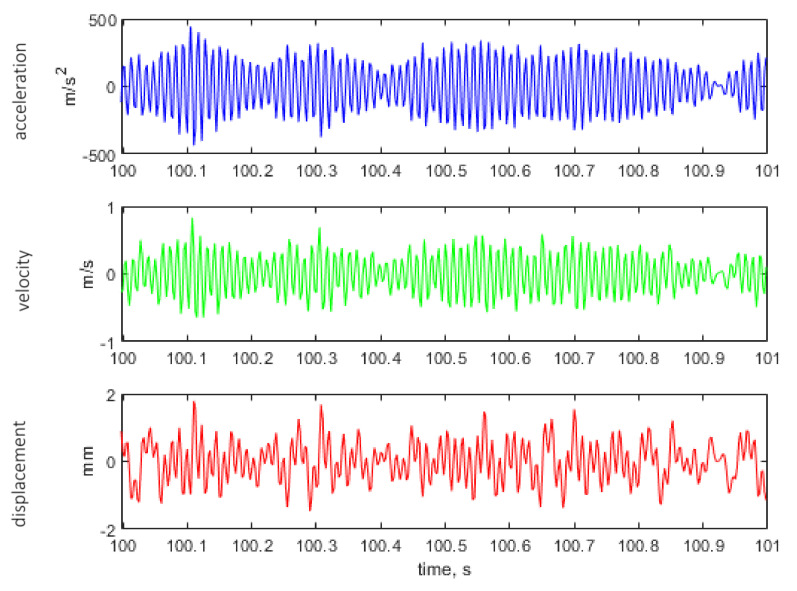
Representative time histories of acceleration measured on additional mass (see Figure 4) and the calculated velocity and displacement for the excitation signal with kurtosis parameter Ku = 3.

**Figure 12:**
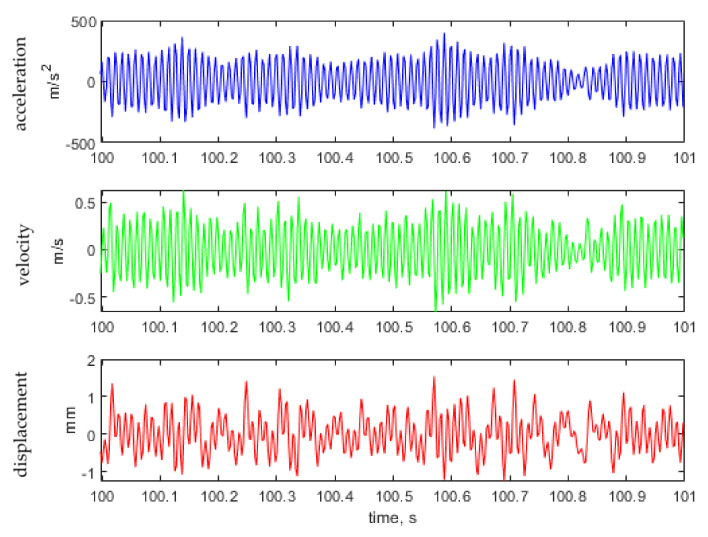
Representative time histories of acceleration measured on additional mass (see Figure 4) and the calculated velocity and displacement for the excitation signal with kurtosis parameter Ku = 4.

**Figure 13:**
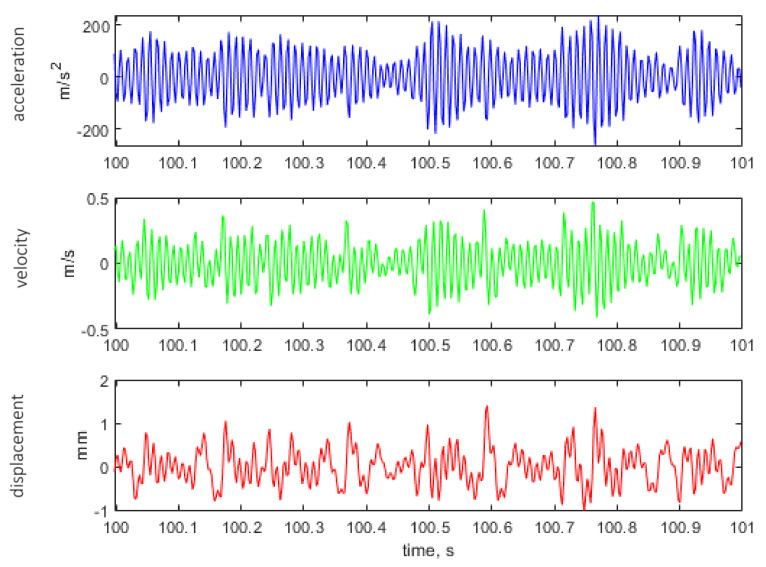
Representative time histories of acceleration measured on additional mass (see Figure 4) and the calculated velocity and displacement for the excitation signal with kurtosis parameter Ku = 5.

Figure 11, Figure 12 and Figure 13 show the representative parts of the acceleration signal and the resultant waveforms of velocity and displacement obtained with the integration methods. The basic acceleration signal $a_{m}\left(t\right)$ was recorded in the measuring system using a piezoelectric accelerometer placed on an additional mass, as presented in Figure 4.

### 3.3. Fracture

**Figure 14:**
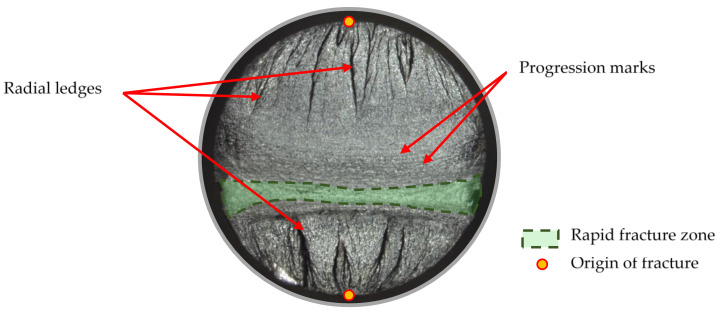
Fatigue fracture with characteristic parts of the crack.

Fatigue cracks, which were observed as a result of two-sided bending, can be classified as double-sided; however, there were also one-sided cracks, which grew from one point on the specimen’s surface. Figure 14 shows a photo of a representative double-sided fatigue crack with characteristic areas and points highlighted. Table 8 lists the fatigue cracks for specimens that obtained a similar fatigue life but under different loading conditions, i.e., for different levels of load and kurtosis. As shown in Table 8, the presented cracks are characterized by fatigue damage on both sides in the vast majority of cases (79%), though single one-sided cracks were also observed (see Table 9). With the increase in kurtosis in the forcing signal on the fracture surface, an intensification of radial ledges was observed. This may be due to the increase in the time history amplitudes with the greater kurtosis values.

For each loading type, without taking into account the loading level, a general relationship in terms of the occurrence of radial ledges can be noted. An increase in radial ledges is observed with a change in the kurtosis parameter of the loading signal. This can be explained by the increase in the proportion of high load amplitudes in the load signal. Fewer progression marks are visible closer to the origin of the crack. There were no significant changes in the occurrence of progression marks with respect to changes in the kurtosis of the loading signal.

## 4. Conclusions and Remarks

The results presented herein indicate the need for very precise experimental fatigue tests. This is especially necessary in cases where random load conditions with a non-Gaussian distribution are used. Test results obtained in this way can be used to verify numerical calculation models, in which various coefficients for correcting the obtained fatigue life are used.

Existing models of fatigue life determination when taking into account the deviation from the normal distribution in order to put emphasis on a comprehensive presentation of the results of fatigue tests were not analyzed. In addition, the following detailed conclusions arose:

- The series of fatigue tests carried out with three kurtosis values showed the clear influence of the kurtosis parameter on the fatigue life obtained. By maintaining the same variance of the load course, the average fatigue life was reduced tenfold when kurtosis was increased from three to four and from four to five.
- The presented fatigue calculations performed in the Ansys Workbench program only correspond to the experimental results obtained for the kurtosis value 3, which is noticeable in Figure 6. This implies the need to take into account the kurtosis parameter and to correct the obtained results of the FEM analysis for the non-Gaussian load.
- There was no correlation between the change in kurtosis and the type of fatigue crack. The majority of cracks were two-sided; one-sided cracks appeared randomly for different loading conditions.
- On the basis of the acceleration signals recorded on the specimen and on the sliding table, different kurtosis values were noted. These result from the occurrence of the first natural frequency of the system at 90 Hz, which is within the PSD load range.

## Figures and Tables

**Table 1:** Experimental loading conditions for kurtosis *Ku* = 3.

Loading $g_{RMS}$	$\mathrm{Amplitude},\;g^{2}/Hz$	Maximum Force N	Maximum Acceleration g
1.1	0.015125	539	5.46
1.2	0.018	588	5.98
1.3	0.021125	637	6.47
1.4	0.0245	684	6.97

**Table 2:** Experimental loading conditions for kurtosis *Ku* = 4.

Loading $g_{RMS}$	$\mathrm{Amplitude},\;g^{2}/Hz$	Maximum Force N	Maximum Acceleration g
0.85	0.009031	547	5.58
1.0	0.0125	643	6.56
1.1	0.015125	708	7.22
1.2	0.018	772	7.87

**Table 3:** Experimental loading conditions for kurtosis *Ku* = 5.

Loading $g_{RMS}$	$\mathrm{Amplitude},\;g^{2}/Hz$	Maximum Force N	Maximum Acceleration g
0.85	0.009031	649	6.62
0.9	0.010125	667	7.01
1.0	0.0125	764	7.79

**Table 4:** Experimental results.

Kurtosis	Loading $g_{RMS}$	Fatigue Life s	Surface Roughness Ra μm	Specimen Diameter mm	Crack Type Single/Double
3.0	1.1	7500	0.342	7.98	Single
	1.1	26,100	0.368	7.97	Single
	1.2	23,100	0.573	8.01	Double
	1.2	900	0.383	7.97	Double
	1.2	12,600	0.303	7.94	Double
	1.3	2460	0.278	7.94	Double
	1.3	4680	0.397	8.01	Double
	1.3	2100	0.212	7.96	Double
	1.4	660	0.446	7.97	Double
	1.4	1200	0.351	7.94	Double
	1.4	1020	0.175	7.97	Double
4.0	0.85	nc	0.367	7.98	---
	1.0	2220	0.294	7.96	Double
	1.0	1440	0.431	7.99	Double
	1.1	1380	0.527	7.97	Single
	1.1	15,660	0.347	7.99	Double
	1.1	660	0.581	8.02	Double
	1.1	1320	0.289	7.98	Double
	1.2	900	0.263	8.00	Double
	1.2	2700	0.388	7.99	Double
	1.2	480	0.268	8.01	Double
5.0	0.85	3660	0.398	7.98	Double
	0.85	15,240	0.496	7.99	Single
	0.85	16,380	0.243	8.00	Single
	1.0	600	0.409	8.01	Double
	1.0	240	0.492	8.00	Double
	1.0	900	0.278	7.96	Double
	0.9	nc	0.296	7.98	---
	0.9	1380	0.411	8.01	Single
	0.9	nc	0.364	7.99	---
	0.9	480	0.297	7.98	Double

nc—no cracks after 8 h (maximum test duration).

**Table 5:** Experimental results and numerical prediction for tested cases with different kurtosis values.

**Loading** **g_RMS_**	Amplitude $g^{2}/Hz$	Experiment Duration, s Ku = 3 $Nf_{exp}$	Experiment Duration, s Ku = 4 $Nf_{exp}$	Experiment Duration, s Ku = 5 $Nf_{exp}$	Numerical Prediction, s $Nf_{calc}$
0.85	0.009031	–	28,800 *	3660 15,240 16,380	88,389
0.9	0.010125	–	–	1380 480 28,800 * 28,800 *	55,476
1.0	0.0125	–	2220 1440	600 240 900	20,071
1.1	0.015125	7500 26,100	1380 15,660 660 1320	–	8414
1.2	0.018	23,100 900 12,600	900 2700 480	–	3804
1.3	0.021125	2460 4680 2100	–	–	1833
1.4	0.0245	660 1200 1020	–	–	932

* experiment stopped after the maximum test time.

**Table 6:** Analysis details.

Quantity.	Global	Neck Region
Nodes	139,507	130,436
Elements	65,391	63,709
Element size	4 mm	1 mm
Refinement level	-	3
Elements Types	HEX20, TET10 (>95%)	

**Table 7:** Modal frequencies of the experimental test rig.

Mode No.	Frequency Hz	Type	Shape
1	90.039	Bending (YZ plane)	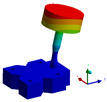
2	90.057	Bending (XY plane)	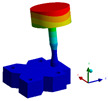
3	207.88	Twisting (Y-axis)	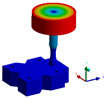
4	1015.5	Complex (YZ plane)	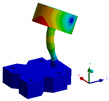
5	1017.9	Complex (XY plane)	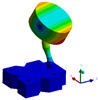
6	1951	Tension (Y-axis)	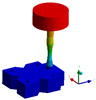

**Table 8:** Presentation of fatigue fractures for the same test duration and different kurtosis values.

	Ku = 3 (Figure 10)	Ku = 4 (Figure 10)	Ku = 5 (Figure 12)
Loading level	1.2 g _RMS_	1.2 g _RMS_	1.0 g _RMS_
Test duration	902 s	904 s	900 s
Total fracture *	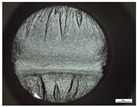	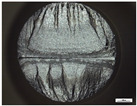	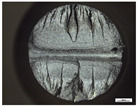

* All photos of fatigue fractures were taken with the same magnification; the scale marked in the lower right corners shows the value of 1000 μm.

**Table 9:** Summary of one-sided fatigue fractures obtained under various load conditions.

	Ku = 3 (Figure 10)	Ku = 4 (Figure 10)	Ku = 5 (Figure 12)
single-sided fracture			
Loading level	1.1 g _RMS_	1.1 g _RMS_	0.85 g _RMS_
Test duration	26,110 s	1380 s	16,380 s
Total fracture * single-sided	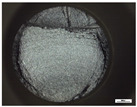	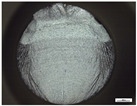	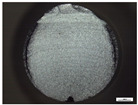

* All photos of fatigue fractures were taken with the same magnification; the scale marked in the lower right corners shows the value of 1000 μm.

## Data Availability

Data sharing not applicable.

## References

[1] Niesłony A., Růžička M., Papuga J., Hodr A., Balda M., Svoboda J. (2012). Fatigue life prediction for broad-band multiaxial loading with various PSD curve shapes. Int. J. Fatigue.

[2] Zheng R., Chen H., He X. (2017). Control method for multi-input multi-output non-Gaussian random vibration test with cross spectra consideration. Chin. J. Aeronaut..

[3] Benasciutti D., Tovo R. (2006). Fatigue life assessment in non-Gaussian random loadings. Int. J. Fatigue.

[4] Gao D.-Y., Yao W.-X., Wu T. (2019). A damage model based on the critical plane to estimate fatigue life under multi-axial random loading. Int. J. Fatigue.

[5] Xu J., Zhang Y., Han Q., Li J., Lacidogna G. (2020). Research on the Scope of Spectral Width Parameter of Frequency Domain Methods in Random Fatigue. Appl. Sci..

[6] Zheng R., Chen H., He X., Zheng W. (2018). Probability distributions control for multi-input multi-output stationary non-Gaussian random vibration test. J. Vib. Control.

[7] Niesłony A., Macha E. (2007). Spectral Method in Multiaxial Random Fatigue.

[8] Celikoglu A., Tirnakli U. (2014). Skewness and kurtosis analysis for non-Gaussian distributions. arXiv.

[9] Kihm F., Ferguson N., Antoni J. (2015). Fatigue Life from Kurtosis Controlled Excitations. Procedia Eng..

[10] Braccesi C., Cianetti F., Lori G., Pioli D. (2009). The frequency domain approach in virtual fatigue estimation of non-linear systems: The problem of non-Gaussian states of stress. Int. J. Fatigue.

[11] Celikoglu A., Tirnakli U. (2015). Comment on “Universal relation between skewness and kurtosis in complex dynamics”. Phys. Rev. E.

[12] Krasil’Nikov A.I. (2013). Class of non-Gaussian distributions with zero skewness and kurtosis. Radioelectron. Commun. Syst..

[13] Kihm F., Rizzi S., Ferguson N., Halfpenny A. (2013). Understanding How Kurtosis Is Transferred from Input Acceleration to Stress Response and It’s Influence on Fatigue Life. Presentedon RASD 2013 (30/06/13–02/07/13). https://www.ocs.soton.ac.uk/index.php/rasdconference/RASD2013/paper/view/1006.

[14] Steinwolf A., Cornelis B., Peeters B., Van Der Auweraer H., Rivola A., Troncossi M. (2019). On the Use of Kurtosis Control Methods in Shaker Testing for Fatigue Damage. J. Test. Evaluation.

[15] Zanellati D., Benasciutti D., Tovo R. (2018). Vibration fatigue tests by tri-axis shaker: Design of an innovative system for uncoupled bending/torsion loading. Procedia Struct. Integr..

[16] Khalij L., Gautrelet C., Guillet A. (2015). Fatigue curves of a low carbon steel obtained from vibration experiments with an electrodynamic shaker. Mater. Des..

[17] Macek W., Owsiński R., Trembacz J., Branco R. (2020). Three-dimensional fractographic analysis of total fracture areas in 6082 aluminium alloy specimens under fatigue bending with controlled damage degree. Mech. Mater..

[18] Niesłony A., Böhm M., Owsiński R. (2020). Formulation of multiaxial fatigue failure criteria for spectral method. Int. J. Fatigue.

[19] Niesłony A., Owsiński R., Dziura A. (2018). Methods of description of random loading in fatigue life calculation. Fourth Huntsville gamma-ray burst symposium.

[20] Kurek A., Kurek M., Koziarska J., Vantadori S., Łagoda T. (2018). Fatigue characteristics of 6082-T6 aluminium alloy obtained in tension-compression and oscillatory bending tests. J. Mach. Constr. Maint.—Probl. Eksploat..

[21] MIL STD 810 | Vibration. CELAB.

[22] Lachowicz C.T., Owsiński R. (2020). Comparative Analysis of Fatigue Energy Characteristics of S355J2 Steel Subjected to Multi-Axis Loads. Materials.

